# Anti-Inflammatory Potential of Wampee (*Clausena lansium* (Lour.) Skeels) Polyphenol Extract in Ulcerative Colitis: Gut Microbiota and TLR4-p38 MAPK/NF-κB Signaling Axis Regulation

**DOI:** 10.3390/foods14040619

**Published:** 2025-02-13

**Authors:** Kaijie Shang, Zhiheng Zhao, Hua Chen, Xiaonan Bian, Xianquan Zhong, Xiaoping Hu, Xue Lin, Lu Wang

**Affiliations:** 1 School of Food Science and Engineering, Hainan University, Haikou 570228, China; 22210832000009@hainanu.edu.cn (K.S.); 21210832000034@hainanu.edu.cn (Z.Z.); chenh330@163.com (H.C.); 22220951350004@hainanu.edu.cn (X.B.); 23210832000023@hainanu.edu.cn (X.Z.); xuelin@hainanu.edu.cn (X.L.); wang@hainanu.edu.cn (L.W.); 2 Key Laboratory of Food Nutrition and Functional Food of Hainan Province, Hainan University, Haikou 570228, China

**Keywords:** wampee, ulcerative colitis, gut microbiota, short chain fatty acids, signaling pathway

## Abstract

The consumption of wampee has traditionally been utilized to alleviate gastrointestinal inflammation and associated disorders; however, its exact mechanism has remained unknown. The aim of this study was to elucidate the therapeutic efficacy and underlying mechanism of wampee polyphenol extract (WPE) in dextran sulfate sodium (DSS)-induced ulcerative colitis (UC). The findings revealed that WPE alleviated diverse symptoms of UC, regulated various inflammatory cytokines, and effectively protected the colon tissue structure and barrier integrity, thereby inhibiting LPS translocation. Moreover, WPE restored the richness and diversity of gut microbiota and optimized its structure at the phylum and genus levels, causing a notable improvement in short- chain fatty acid (SCFA) metabolism, particularly acetic acid, propionic acid, and butyric acid. Consequently, WPE was demonstrated to effectively suppress the LPS-induced TLR4-p38 MAPK/NF-κB signaling pathway by modulating gut microbiota and SCFA metabolism. These findings provided a theoretical basis for the use of wampee as a potential functional natural food for UC.

## 1. Introduction

Wampee (*Clausena lansium* (Lour.) Skeels) is an evergreen small tree belonging to the Rutaceae family, indigenous to southern China, and has been widely introduced into other Asian countries, such as Vietnam and the Philippines [1]. The flavor profiles of the peel, pulp and seeds of wampee fruit vary distinctively, and all three parts are edible and rich in bioactive substances; however, the peel and seeds are often discarded as waste due to varying dietary preferences and customs [1,2].

Recent researches have mainly focused on identifying the bioactive constituents in wampee, as well as analyzing and applying their biological properties. Chen et al. [3] investigated the phenolic profile and *α*-glucosidase inhibitory activity of wampee peel and pulp during an in vitro digestion process. Wang et al. [4] extracted the essential oil from wampee seeds and combined it with chitosan to create a composite film that can effectively prolong the refrigerated storage duration of golden pompano filets. Zhang et al. [5] isolated, purified, and identified various alkaloids in the branches and leaves of wampee, and subsequently assessed their inhibitory ability against *Phytophthora capsici* Leonian. Zhou et al. [6] reported the inhibitory effects of wampee polyphenols on pectinase, mediated through interactions with the enzyme and its substrates, namely, cell wall pectic polysaccharides. Furthermore, Shu et al. [7] explored the therapeutic potential of wampee extract for treating metabolic cardiomyopathy in mice.

Ulcerative colitis (UC), a prototypical type of inflammatory bowel disease (IBD), is distinguished by clinical manifestations including diarrhea, abdominal pain, hematochezia, and a tendency to recurrence [8]. As the incidence and prevalence rates escalate annually, UC has emerged as a formidable global health challenge for human beings [9]. Although the precise pathogenesis remains unclear, there is some evidence suggesting a correlation with intestinal microbial dysbiosis and intestinal barrier dysfunction [8].

Due to its rich content of anti-inflammatory compounds, including polyphenols and carbazole alkaloids, wampee is usually esteemed as a medicinal resource in traditional medicine for the prevention or treatment of various inflammatory disorders, such as pharyngitis, bronchitis, hepatitis, and gastrointestinal inflammation [1,10]. Despite the frequent application of wampee in traditional medicine for the prevention and treatment of gastrointestinal disorders, there is still a lack of studies regarding the underlying mechanisms by which wampee alleviates these conditions, particularly UC, and the associated functional pathways in organisms.

The objective of this study is to delineate the therapeutic efficacy and explore potential mechanisms of wampee polyphenol extract (WPE) in a dextran sulfate sodium (DSS)-induced UC mouse model by conducting a thorough analysis of clinical symptoms, biochemical parameters, histopathological alteration in the colon, and expression of key proteins in metabolic pathways, as well as the compositions of the colonic microbiota and the metabolic products of the gut microbiota, especially short chain fatty acids (SCFAs). This research not only provides a scientific rationale for the use of WPE in the treatment of gastrointestinal disorders, particularly UC, but also establishes a foundation for the future development and utilization of wampee fruit.

## 2. Materials and Methods

### 2.1. Chemicals and Reagents

NaOH, HCl, and ethanol were purchased from Xilong Scientific Co., Ltd. (Shantou, China). AB-8 macroporous resin, FeCl3, 1,1-diphenyl-2-picrylhydrazyl (DPPH), and 2,4,6-tris(2-pyridyl)-s-triazine (TPTZ) were procured from Shanghai Aladdin Biochemical Technology Co., Ltd. (Shanghai, China). Trolox, Folin- Clocalteu’s phenol reagent, and salicylazosulfapyridine (SASP) were sourced from Shanghai Yuanye Biochemical Technology Co., Ltd. (Shanghai, China). DSS (36–50 kDa) was obtained from Sigma-Aldrich (St. Louis, MO, USA). All standards, including kaempferol, chlorogenic acid, isoquercetin, dihydromyricetin, quercetin, luteolin, rutin, naringenin, quercitrin, and myricetin, were of high-performance liquid chromatography (HPLC)- grade purity (>97%) and purchased from Shanghai Macklin Biochemical Co., Ltd. (Shanghai, China). The commercial kits for interleukin (IL)-1*β*, IL-6, tumor necrosis factor (TNF)-*α*, IL-10, and lipopolysaccharide (LPS) were acquired from MultiSciences Biotech Co., Ltd. (Hangzhou, China). The qualitative detection kit for fecal occult blood was sourced from Shanghai Enzyme-linked Biochemical Technology Co., Ltd. (Shanghai, China).

### 2.2. Preparation of WPE

Fresh fruits of Yongxing Dajixin wampee, with 90% maturity (102 ± 5 days after flowering), were provided by the Yongxing Plantation Base in Hainan Province. After removing seeds, the lyophilized fruit powder was prepared. With a modified version of a previously reported method [2], the freeze-dried powder was hydrolyzed with 2 M NaOH for 4 h and immediately acidified to a pH of 2 using 6 M HCl. Subsequently, an equal volume of ethyl acetate was introduced for extraction, and this procedure was repeated 3 times. Following the combination of the ester phases, the ethyl acetate was evaporated and removed. Finally, the resultant crude extract was redissolved in distilled water, purified through an AB-8 macroporous resin column with 90% ethanol elution, and then lyophilized again to obtain WPE.

### 2.3. Characterization of Phytochemical Compounds and Antioxidant Activities

#### 2.3.1. Determination of Total Phenolic Content (TPC) and Total Flavonoid Content (TFC)

Following the procedure described by Chen et al. [3], the TPC and TFC of WPE was determined using the Folin- Ciocalteu method and an aluminum trichloride colorimetric assay, expressed in gallic acid equivalents (GAEs) and rutin equivalents (REs) respectively. Quantification of the TPC was performed using the regression curve (Y = 0.0136X + 0.1679, R^2^ = 0.9996) derived from the absorbance at 765 nm, and the TFC was calculated using the regression curve (Y = 0.0008X + 0.0190, R^2^ = 0.9971) obtained from the absorbance at 510 nm.

#### 2.3.2. Quantification of Major Phenolic Compounds

In accordance with the methodologies detailed by Lin et al. and Chen et al. [2,3], the quantification of 10 primary phenolic constituents in WPE was performed using HPLC. An Agilent 1200 HPLC system, equipped with a 250 mm × 4.6 mm C18 column (5 μm, Agilent, Santa Clara, CA, USA), was used for the separation and quantification. The column temperature was maintained at 30 °C, with a flow rate of 0.8 mL/min and a detection wavelength of 280 nm. Mobile phase A was 0.1% formic acid solution, whereas mobile phase B consisted of acetonitrile with an addition of 0.1% formic acid. The elution gradient was programmed as follows: 85% A for 6 min, followed by a gradient decrease to 80% A over 4 min, then to 75% A over 10 min, to 65% A over 10 min, to 50% A over 10 min, and to 20% A over 15 min, and finally returning to 85% A over 5 min.

#### 2.3.3. Evaluation of In Vitro Antioxidant Activities

The DPPH radical scavenging ability and ferric reducing antioxidant power (FRAP) were assessed using the established protocols [11,12] with slight modifications. In the DPPH assay, 50 μL of the sample and various concentrations of Trolox were added to 400 μL of DPPH solution (100 μM in methanol), followed by incubation in the dark for 30 min. The DPPH scavenging capacity was quantified based on the regression curve (Y = −0.2283X + 0.4911, R^2^ = 0.9931) and the absorbance at 517 nm, which was expressed as mM of Trolox equivalent per g of WPE dry weight (mM TE/g DW). In the FRAP assay, a ratio of 10:1:1 (*v*/*v*/*v*) of acetate buffer solution (300 mM, pH = 3.6), FeCl_3_ solution (20 mM), and TPTZ solution (10 mM) was mixed and incubated at 37 °C for 30 min to prepare the FRAP reagent. Subsequently, 50 μL of the sample and different concentrations of Trolox were added into 900 μL of FRAP reagent, and kept in the dark for 30 min. According to the regression curve (Y = 0.6146X − 0.0157, R^2^ = 0.9968) and the absorbance at 593 nm, the FRAP assay results were expressed as mM of Trolox equivalent per g of WPE dry weight (mM TE/g DW).

### 2.4. Animal Experiment

The animal experiment was conducted in accordance with the method described by Liu et al. [13] with minor modifications. A total of 35 male mice (C57BL/6, specific pathogen-free) were purchased from Beijing Vital River Laboratory Animal Technology Co., Ltd. (Beijing, China) and maintained in a standardized environment (25 ± 1 °C, 55 ± 10% humidity, 12 h light/dark cycle). Following a 7 d acclimatization period, the mice were divided into the following groups: a normal control group (NC, *n* = 7), an untreated group (DSS, *n* = 7), a low-dose treatment group (LD, *n* = 7), a high-dose treatment group (HD, *n* = 7), and a positive control group (SASP, *n* = 7). With the exception of the NC group, all mice were administered 3.2% (*w*/*v*) DSS in their drinking water for an 8 d period to induce UC. Subsequently, the LD, HD, and SASP groups were orally administered 100 mg/kg, 400 mg/kg, and 100 mg/kg of WPE, WPE, and SASP (a drug commonly used in the clinical treatment of UC), respectively, daily for a 11 d period via oral gavage. The NC and DSS groups were provided with sterile water daily. The mice’s weight, fecal characteristics, and occult blood were continuously monitored every day throughout the entire process, and the disease activity index (DAI) was calculated based on the previous method [13]. At the end of treatment, the mice were sacrificed by cervical dislocation after retro-orbital blood collection, and their colons and spleens were removed and measured.

### 2.5. Histopathological Analysis

The distal colon specimens were first rinsed with cold PBS and then immersed in a tissue fixative solution (Servicebio Technology Co., Ltd., Wuhan, China). Subsequently, the colon specimens were sectioned using an RM2016 pathological microtome (Leica Instruments Co., Ltd., Shanghai, China) and dewaxed, and then stained with hematoxylin-eosin (H&E) for histological assessment based on a previously used method [14].

### 2.6. Immunohistochemical Analysis

The expression of target proteins ZO-1 and occludin were assessed through immunofluorescence staining. Briefly, after the dewaxing of the paraffin sections from the colon specimens, antigen retrieval was conducted. The sections were allowed to cool naturally before being rinsed with cold PBS 3 times and subsequently treated with a blocking solution for 30 min. Subsequently, the primary antibody was applied and incubated at 4 °C overnight, followed by a washing step with cold PBS 3 times. The secondary antibody was then added and incubated for 50 min at room temperature, followed by a washing step with cold PBS 3 times. Finally, the samples were stained and observed using a NIKON ECLIPSE C1 upright fluorescence microscope (Nikon Co., Tokyo, Japan). The mean gray values obtained through Image J software (Version 1.8.0.112, National Institutes of Health, Bethesda, MD, USA) were used to quantify the relative expression of ZO-1 and occludin.

### 2.7. Serum Biochemical Analysis

Blood samples were collected via retro-orbital sampling, and serum was isolated by centrifugation (3000× *g*, 15 min, 4 °C) after 2 h of standing at room temperature. The contents of IL-1*β*, IL-6, TNF-*α*, IL-10, and LPS in the serum were measured with the corresponding commercial kits.

### 2.8. Western Blotting (WB) Analysis

The expression levels of the target proteins Toll-like receptor 4 (TLR4), p38 mitogen-activated protein kinase (p38 MAPK), and nuclear factor-κB p65 (NF-κB p65) were determined via WB analysis. Briefly, after separation, proteins were blocked with 5% skim milk at room temperature for 30 min. The respective primary antibody was then applied and incubated overnight at 4 °C. An additional 30 min of room temperature incubation was conducted with the secondary antibodies. Finally, the immunoblotting bands were visualized using chemiluminescent reagents and a chemiluminescence imaging system (Clinx Science Instruments Co., Ltd., Shanghai, China), and the band intensities were quantified using Image J software.

### 2.9. 16S rDNA Gene Sequencing and Quantification of SCFAs

Cecal contents were collected, immediately frozen in liquid nitrogen, and subsequently subjected to 16S rDNA gene sequencing and SCFAs analysis. Briefly, after the extraction of total microbial DNA, PCR amplification was conducted using Pfu DNA Polymerase (TransGen Biotech Co., Ltd., Beijing, China). Fluorescence quantification was performed using the Quant-iT PicoGreen dsDNA Assay Kit (Thermo Fisher Scientific Inc., Shanghai, China). Subsequently, sequencing was performed on a NovaSeq sequencer (Illumina, San Diego, CA, USA) with the NovaSeq 6000 SP Reagent Kit (500 cycles).

In addition, an appropriate amount of cecal content was mixed with 500 μL of water and centrifuged at 12,000 rpm at 4 °C for 10 min. Subsequently, 200 μL of the supernatant was collected and combined with 100 μL of 15% phosphoric acid solution, 20 μL of 4-methylvaleric acid solution (375 μg/mL), and 280 μL of ether. This mixture was then centrifuged under the same conditions to obtain the supernatant. The analysis of SCFAs was conducted using a Thermo Trace 1310 gas chromatograph (GC) (Thermo Fisher Scientific Inc., Waltham, MA, USA) in conjunction with a Thermo ISQ LT mass spectrometer (MS) (Thermo Fisher Scientific Inc., Waltham, MA, USA). The sequencing data were processed and analyzed employing the BioNovoGene Cloud Platform (Version 2.0), which is based on the QIIME 2 framework and uses the R programming language.

### 2.10. Statistical Analysis

The results were expressed as the mean ± standard deviation (SD) of at least triplicate experiments. One-way analysis of variance (ANOVA) with Tukey’s multiple comparison test and Spearman’s correlation analysis were employed using SPSS 26.0 (IBM, Armonk, NY, USA) software. The correlation heatmap was plotted using the R programming language.

## 3. Results

### 3.1. Analysis of Polyphenol Composition and Antioxidant Capacity of WPE

The TPC, TFC, antioxidant activities, and contents of major phenolic compounds quantified by HPLC of WPE are summarized in Table S1. The TPC and TFC of WPE were 372.50 ± 13.62 mg GAE/g DW and 189.39 ± 7.49 mg RE/g DW, respectively. Moreover, WPE exhibited notable antioxidant activity in DPPH and FRAP assays, with values of 13.89 ± 0.53 and 14.83 ± 0.48 mM TE/g DW, respectively. He et al. [9] prepared a sweet tea extract (STE) with TPC of 200.68 ± 5.68 mg GAE/g DW and TFC of 38.95 ± 0.43 mg RE/g DW. The STE possessed an effective outcome in the treatment of DSS-induced UC. Similarly, a dry powder of grape seeds prepared by Pistol et al. [15] exhibited a TPC of 55.67 mg GAE/g DW and an antioxidant activity of 5.05 mM TE/g DW in DPPH assay, and this powder demonstrated substantial efficacy in the treatment of UC. Therefore, the high levels of TPC, TFC, and antioxidant activities of WPE indicated it could have potential therapeutic efficacy for the UC treatment.

A total of 10 major phenolic compounds were quantified via HPLC (Table S1). Kaempferol was the highest (37,196.89 ± 328.72 μg/g DW) in WPE, followed by chlorogenic acid (32,527.43 ± 348.66 μg/g DW), isoquercetin (32,369.78 ± 279.84 μg/g DW), dihydromyricetin (24,668.86 ± 210.53 μg/g DW), quercetin (19,777.20 ± 263.10 μg/g DW), luteolin (19,166.84 ± 166.28 μg/g DW), rutin (18,624.70 ± 258.32 μg/g DW), and so on. Kaempferol, a natural flavonoid, exhibits limited oral availability but does not require absorption into the bloodstream circulatory system to induce its biological effects, and it has been demonstrated to alleviate intestinal angiogenesis and mitigate the symptoms of DSS-induced colitis by regulating the colonic microbiota composition [16,17]. Chlorogenic acid, a widely consumed antioxidant polyphenol in the human diet, has been proven to suppress the mRNA expression of colorectal inflammatory protein 2 and IL-1*β* induced by DSS treatment, and to attenuate the secretion of IL-8 in response to TNF-*α* and H_2_O_2_ stimulation [18]. Both isoquercetin and dihydromyricetin have been recognized as having potential therapeutic value for treating DSS-induced colitis. Duan et al. [19] suggested that the kaempferol, quercetin, and luteolin present in Wumei Wan may contribute to ameliorating DSS-induced colitis. The therapeutic potential of these compounds was linked to their ability to modulate the inflammation-related signaling pathways through the regulation of gut microbiota [20,21]. Furthermore, previous study has demonstrated that rutin possesses the capacity to reduce intestinal inflammation by diminishing pro-inflammatory cytokines and inhibiting the NF-κB and MAPK signaling pathway [22]. Therefore, WPE is likely to have an effective outcome in the treatment for UC by regulating the relevant inflammatory pathways through these key polyphenols.

### 3.2. The Effect of WPE on UC Symptoms and Colon Injury

**Figure 1:**
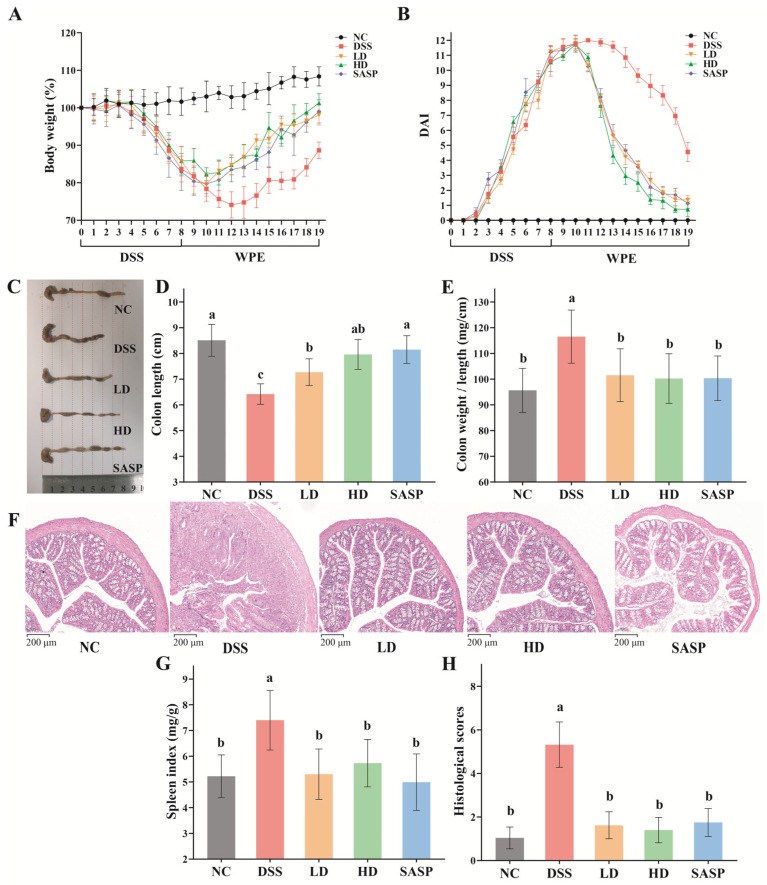
Effect of WPE on disease symptoms and colonic damage. (**A**) Changes in daily body weight; (**B**) changes in DAI score; (**C**) representative image of colon length; (**D**) colon length; (**E**) ratio of colon weight to length; (**F**) H&E—stained images of colon tissues; (**G**) spleen index (ratio of spleen weight to body weight); (**H**) histological score. All data are expressed as mean ± SD (*n* = 7). Bars with different lowercase letters indicate statistically significant differences (*p* < 0.05).

Compared to the NC group, DSS led to a rapid drop in body weight among all other groups (Figure 1A). The weight loss process in the DSS group was the most prolonged, with the nadir occurring on the 12th day at 74.08 ± 3.27% of the initial body weight. The lowest points in body weight for the LD, HD, and SASP groups were observed on the 10th day, with respective values of 79.82 ± 2.86%, 82.27 ± 1.76%, and 79.88 ± 2.28%. There were no significant differences in the nadir body weights among the three groups, all of which were significantly greater than that of the DSS group (*p* < 0.05). After the 11 d treatment period, the body weights of the LD, HD, and SASP groups reverted to 98.04 ± 2.64%, 101.27 ± 2.48%, and 99.03 ± 3.10%, respectively. Similarly, no significant differences were found among the three groups, with all groups displaying notably higher body weights than the DSS group (88.66 ± 2.20%, *p* < 0.05). Furthermore, the administration of WPE and SASP markedly accelerated the decline in DAI (Figure 1B). After the 11th day, the DAI values of each treatment group were notably decreased relative to those of the DSS group (*p* < 0.01). From the 13th to the 16th days, the DAI of the HD group was notably lower, compared to the LD group (*p* < 0.05). However, no significant differences in DAI were observed among the LD, HD, and SASP groups at the end of the treatment period. It was evident that WPE significantly promoted the recovery of body weight and DAI in mice. Although high-dose WPE exhibited better efficacy at a particular stage of treatment, no evident dose-dependence was observed during the 11-day treatment process.

Reduction in colon length, augmentation of the colon weight-to-length ratio, and elevation in the spleen index are commonly considered typical symptoms of UC. From Figure 1C,D, it can been seen that WPE and SASP attenuated the reduction in colon length resulting from DSS-induced UC. The colon length of the NC group (8.51 ± 0.62 cm) and all treatment groups was significantly higher than that of the DSS group (6.42 ± 0.40 cm). No significant differences were observed between the LD group (7.27 ± 0.52 cm), the HD group (7.96 ± 0.58 cm), and the SASP group (8.15 ± 0.54 cm). Moreover, as shown in Figure 1E, the colon weight-to-length ratio did not display any significant differences among the NC (95.62 ± 8.56 mg/cm), LD (101.54 ± 10.25 mg/cm), HD (100.23 ± 9.65 mg/cm), and SASP groups (95.62 ± 8.56 mg/cm), while the DSS group (116.50 ± 10.32 mg/cm) exhibited a significantly higher one (*p* < 0.05). Similarly, the spleen indices of the NC (5.22 ± 0.83 mg/g), LD (5.30 ± 0.98 mg/g), HD (5.73 ± 0.92 mg/g), and SASP groups (4.99 ± 1.10 mg/g) did not show significant variations and were all lower than the DSS group (7.40 ± 1.16 mg/g, *p* < 0.05).

Colonic histological examination (Figure 1F,H) revealed that the colon epithelial tissue of the NC- group mice was sound, with a normal distribution of goblet cells and intact crypt structures. Conversely, the colonic epithelial structure of DSS- group mice displayed severe damage, characterized by varying degrees of inflammatory cell infiltration, proliferation of fibrous tissue, loss of goblet cells, and alteration in colonic crypt morphology and basal membrane. However, the colon tissue of the mice treated with WPE and SASP exhibited relative integrity, with minimal damage and only minor inflammatory infiltration. Consistent with these observations, the histopathological score of the DSS group was significantly higher (*p* < 0.01).

### 3.3. The Effect of WPE on the Colonic Epithelial Barrier

**Figure 2:**
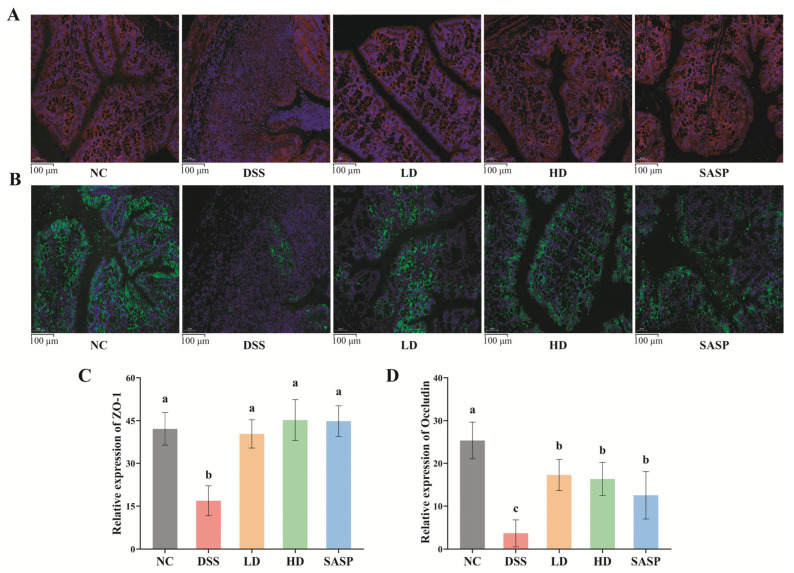
Effect of WPE on the expression of TJ proteins (ZO-1 and occludin). (**A**) Immunofluorescence staining of ZO-1; (**B**) immunofluorescence staining of occludin; (**C**) relative expression of ZO-1; (**D**) relative expression of occludin. Data are expressed as mean ± SD (*n* = 5). Bars with different lowercase letters indicate statistically significant differences (*p* < 0.05).

The expression levels of ZO-1 and occludin within the colon epithelium were evaluated via immunofluorescence staining. ZO-1 was visualized in red (Figure 2A), occludin in green (Figure 2B), and the nucleus in blue (Figure 2A,B). The dim and scatter red and green staining patterns indicated a marked reduction in the expression of ZO-1 and occludin in the intestinal epithelium of DSS-induced UC mice. The dispersed blue dots denoted the destruction of goblet cells and the presence of inflammatory infiltration. It was evident that WPE and SASP treatments have mitigated this trend and restored the expression of ZO-1 and occludin. Correspondingly, the relative expressions of the two proteins in the LD, HD, and SASP groups were significantly higher than those in the DSS group, and no significant difference was observed in the expression of ZO-1 among all the treatment groups and the NC group (Figure 2C,D). Intestinal epithelial cells form a robust physical barrier through tight junctions (TJs), which effectively prevent microorganisms in the intestinal tract from triggering immune responses [23]. TJs are composed of transmembrane proteins like occludin and cytoplasmic scaffold proteins such as ZO-1. It was reported that mice with UC exhibited a damaged intestinal epithelial barrier [8]. Obviously, WPE played a positive role in repairing the colonic epithelial barrier by enhancing the expression of the TJs proteins ZO-1 and occludin.

### 3.4. The Regulation of WPE for Cytokines Secretion and the Expression of Key Proteins in Metabolic Pathways

**Figure 3:**
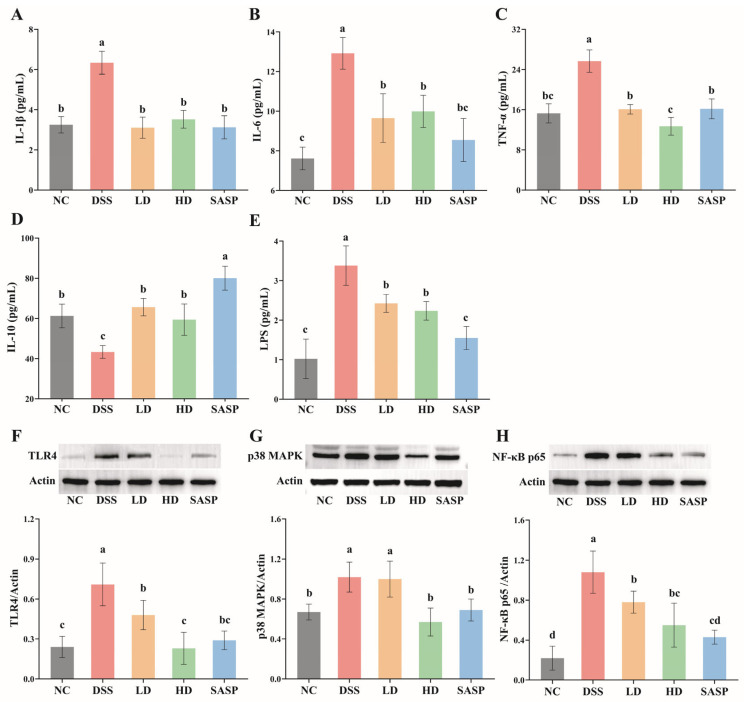
Effect of WPE on the inflammatory cytokines, LPS, and key proteins related to the TLR4-p38 MAPK/NF-κB signaling pathway in serum. (**A**–**C**) The expressions of pro-inflammatory cytokines IL-1*β*, IL-6, and TNF-*α*; (**D**) the expressions of anti-inflammatory cytokine IL-10; (**E**) the contents of LPS; (**F**–**H**) the expressions of TLR4, p38 MAPK, and NF-κB p65 proteins and their corresponding WB images. Data are expressed as mean ± SD (*n* = 5). Bars with different lowercase letters indicate statistically significant differences (*p* < 0.05).

The expression levels of cytokines IL-1*β*, IL-6, TNF-*α*, and IL-10 in the serum of mice across each group are shown in Figure 3A–D. The IL-1*β* concentration in serum from the DSS- group mice was significantly higher (6.37 ± 0.52 pg/mL), and there were no significant differences among the treatment groups and the NC group (LD: 3.10 ± 0.48 pg/mL, HD: 3.52 ± 0.40 pg/mL, SASP: 3.13 ± 0.53 pg/mL, NC: 3.25 ± 0.37 pg/mL). Similarly, the expression level of IL-6 in the mice from the DSS group significantly increased (12.92 ± 0.73 pg/mL), and no significant difference was observed among the treatment groups (LD: 9.65 ± 1.12 pg/mL, HD: 9.99 ± 0.74 pg/mL, SASP: 8.55 ± 0.99 pg/mL), as well as between the SASP group and the NC group (7.61 ± 0.52 pg/mL). DSS-induced UC also led to a notable increase in TNF-*α* expression (NC: 15.28 ± 1.73 pg/mL, DSS: 25.68 ± 2.03 pg/mL). This trend was attenuated by WPE and SASP treatments, and the high-dose WPE resulted in a more favorable result (LD: 16.09 ± 0.87 pg/mL, HD: 12.71 ± 1.61 pg/mL, SASP: 16.18 ± 1.80 pg/mL). Moreover, the concentration of IL-10 in the serum in the DSS- group mice was notably reduced, with a value of 43.32 ± 2.85 pg/mL. There were no significant differences in the expression levels of IL-10 among the NC (61.25 ± 5.36 pg/mL), LD (65.67 ± 3.99 pg/mL), and HD (59.42 ± 7.12 pg/mL) groups. And IL-10 concentration in the SASP group (80.06 ± 5.43 pg/mL) was the highest. Experimental UC was characterized by an imbalance between pro-inflammatory and anti-inflammatory cytokines [24]. IL-1*β*, IL-6, and TNF-*α* were recognized as representative pro-inflammatory cytokines, while IL-10, secreted by regulatory T (Treg) cells, was regarded as an anti-inflammatory cytokine with a protective effect against UC [8]. It was obvious that WPE extensively inhibited the expression of various pro-inflammatory cytokines and enhanced the expression of an anti-inflammatory cytokine.

In addition, the DSS- group mice exhibited the highest LPS concentration in serum, amounting to 3.38 ± 0.45 pg/mL (Figure 3E). In WPE treatment groups, there was significant alleviation in LPS translocation (LD: 2.43 ± 0.21 pg/mL, HD: 2.24 ± 0.21 pg/mL, DSS: 3.38 ± 0.45). Furthermore, the LPS concentration of the SASP group (1.55 ± 0.27 pg/mL) returned to the normal level (1.02 ± 0.46 pg/mL) observed in the NC group. UC often results in the disruption of the intestinal epithelial barrier, which commonly leads to the translocation of LPS from the intestinal lumen to the circulatory system. Consequently, LPS serves as an important marker for assessing the integrity of the intestinal epithelial barrier [9]. The massive translocation of LPS indicated that the intestinal epithelial barrier of mice in the DSS group was compromised, and the administration of WPE contributed to the mitigation of this impairment. And this result was in concordance with the observation in immunohistochemistry analyses.

Moreover, translocated LPS can act as a ligand to bind to TLR4, thereby activating the NF-κB signaling pathway, which is modulated by diverse, independent mechanisms involving p38 MAPK [25,26,27]. p38 MAPK, a critical member of the MAPK family, plays a key role in producing inflammatory mediators and promoting the expression of genes encoding pro-inflammatory molecules in the classic MAPK signal cascade [28]. Such a TLR4-p38 MAPK/NF-κB signaling axis activated by LPS can cause abnormal expressions of IL-6, IL-1*β*, and TNF-*α* [28]. Thus, WPE may to some extent exert an inhibitory effect on the LPS-induced TLR4-p38 MAPK/NF-κB signaling pathway.

To validate this conjecture, a WB assay was employed to quantify the expression levels of TLR4, p38 MAPK, and NF-κB p65 in the serum from each murine group, with the results illustrated in Figure 3F–H. Obviously, the DSS- group mice displayed the highest relative expression of TLR4, followed by the LD group. No significant differences were observed among the NC, HD, and SASP groups. Although the relative expression of p38 MAPK did not significantly differ between the DSS and LD groups, it notably decreased in the HD group. The expression pattern of NF-κB p65 paralleled that of TLR4, with the DSS group exhibiting the highest relative level, followed by the LD and HD groups. In comparison to the DSS group, the treatment of low-dose WPE resulted in a reduction in the relative expression levels of TLR4, P38 MAPK, and NF-κB p65 by 32.39%, 1.96%, and 27.78%, respectively. Nevertheless, high-dose WPE treatment led to a more pronounced downregulation of TLR4, p38 MAPK, and NF-κB p65 by 67.61%, 44.12%, and 49.07%, respectively. It is evident that the expression of pro-inflammatory cytokines in murine models can be regulated by WPE through the inhibition of the LPS-activated TLR4-p38 MAPK/NF-κB signaling pathway. Moreover, this inhibitory ability of WPE exhibited an obvious dose-dependent relationship.

Furthermore, LPS-activated TLR4 promotes the innate immune and inflammatory response through the NF-κB signaling pathway, typically leading to an increased spleen index [13,29]. In the present study, TLR4 expression and spleen index were significantly reduced in all treatment groups compared to the DSS group, consistent with the above findings. However, the HD group showed significantly lower TLR4 expression than the LD group, while no significant difference in spleen index was observed between the two groups. Similarly, Liu et al. [13] documented inconsistencies between the size relationship of the spleen index and the phosphorylation of the NF-κB in TLR4/NF-κB signaling pathway across different groups in a 16 d study of six tea water extracts as treatments of UC.

### 3.5. The Effect of WPE on the Structure of Gut Microbiota

**Figure 4:**
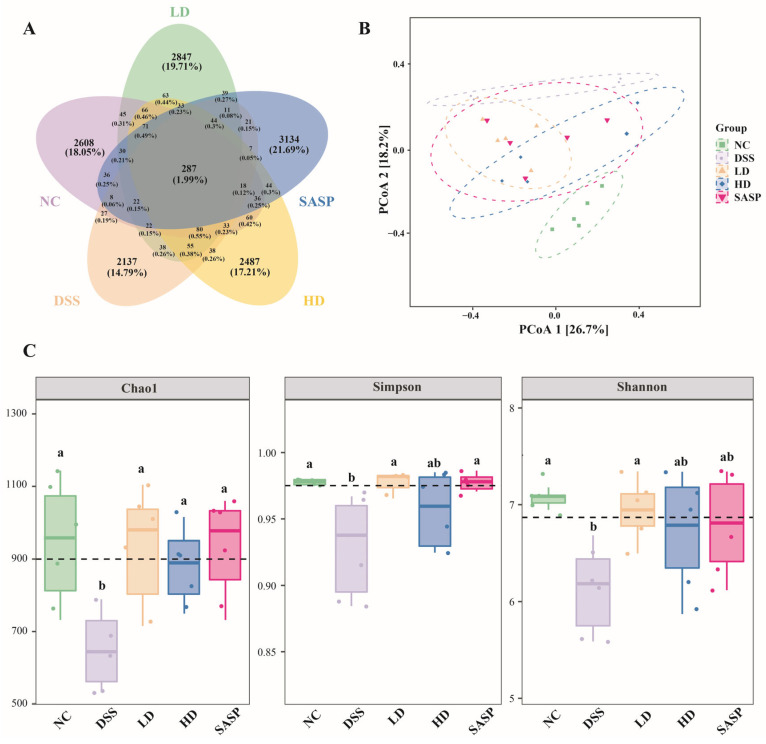
Effect of WPE on gut microbiota diversity. (**A**) Venn diagram of OTU; (**B**) *β* diversity at the OTU level; (**C**) *α* diversity at the OTU level (richness: Chao1 index; diversity: Simpson index and Shannon index). Data are expressed as mean ± SD (*n* = 5). Bars with different lowercase letters indicate statistically significant differences (*p* < 0.05).

Much evidence supports the notion that colitis and inflammation are closely linked to the equilibrium of gut microbiota [30]. 16S rDNA gene sequencing was used to elucidate the impact of WPE on the gut microbiota composition of UC mice. Across the 25 fecal samples (*n* = 5 per group), a total of 14,447 operational taxonomic units (OTUs) were identified, with 287 OUTs shared among all five groups (Figure 4A). In the NC, LD, HD, and SASP groups, 2608, 2847, 2487, and 3134 unique OTUs were observed, respectively. The DSS group had the fewest unique OTUs, totaling 2137. *β* diversity analysis has been a prevalent tool for assessing microbiota profile discrepancies, whereas *α* diversity analysis is commonly used to indicate the richness (Chao1 index) and diversity (Simpson index and Shannon index) of the gut microbiota [31]. *β* diversity was analyzed at the OTU level via principal coordinate analysis (PCoA) as depicted in Figure 4B. It is apparent that the distance between the NC and DSS groups was the farthest, indicating the greatest dissimilarity between these two groups. Additionally, every treatment group displayed a trend towards the NC group. Particularly, the HD group exhibited minimal overlap with the DSS group and was generally closer to the NC group, suggesting that the perturbation in the gut microbiota of UC mice after WPE treatment was attenuated. Moreover, *α* diversity at the OTU level showed similar results (Figure 4C). Across multiple characterization methods, employing the Chao1 index, the Simpson index, and the Shannon index, the DSS group displayed a consistent reduction in *α* diversity compared to the NC group, whereas no significant differences were observed between the treatment groups and the NC group. These findings demonstrated the efficacy of WPE in the loss of gut microbiota diversity in UC mice.

**Figure 5:**
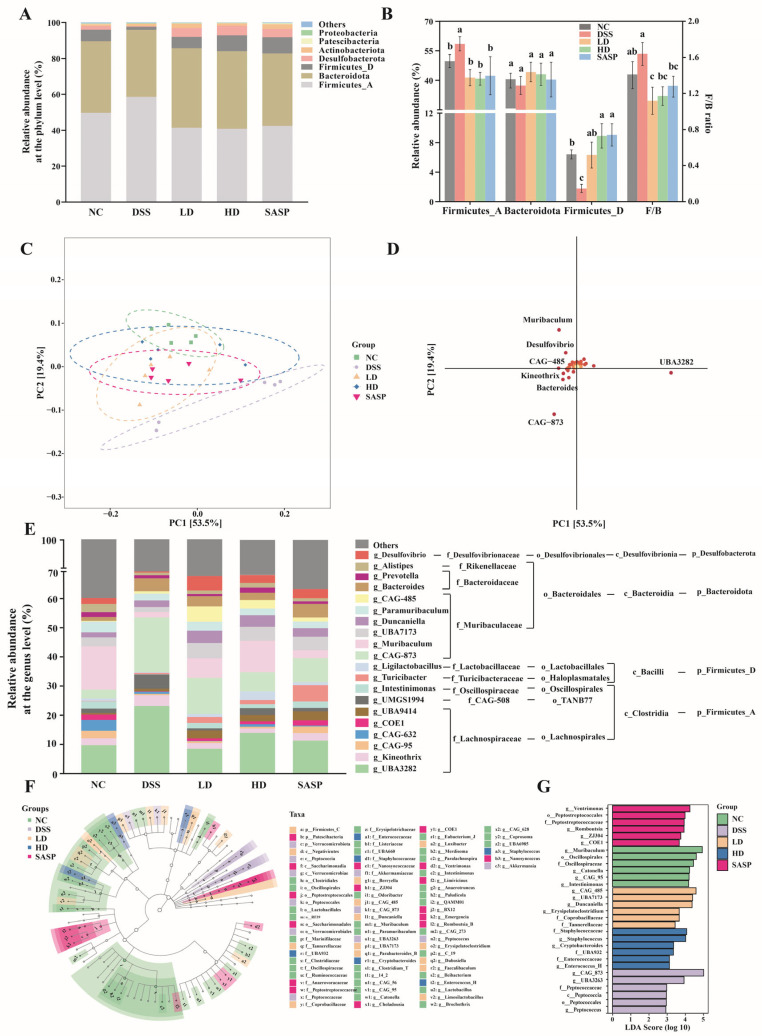
Effect of WPE on gut microbiota structure. (**A**) Relative abundance of gut microbiota at the phylum level; (**B**) relative abundance and ratio of Firmicutes and Bacteroidota; the score plot (**C**) and loading plot (**D**) of PCA at the genus level; (**E**) relative abundance and biological classification of the top 20 gut microbiota at the genus level; LEfSe (**F**) and its influencing factors (**G**). Data are expressed as mean ± SD (*n* = 5). Bars with different lowercase letters indicate statistically significant differences (*p* < 0.05).

The composition of gut microbiota at the phylum level is illustrated in Figure 5A. The seven dominant microbiota (including two subphyla of Firmicutes) comprising a total relative abundance over 99% in each group were Firmicutes_A, Bacteroidota, Firmicutes_D, Desulfobacterota, Actinobacteriota, Patescibacteria, and Proteobacteria. Notably, Firmicutes_A, Bacteroidota, and Firmicutes_D emerged as the most predominant microbial phyla (with a total relative abundance exceeding 90%). Prevalent studies have identified a consistent pattern in both humans and murine gut microbiomes, where approximately 90% of the microbiome is composed of Firmicutes and Bacteroidota [32], aligning with our results. Not only are Firmicutes and Bacteroidota dominant in the intestine, their proportion also is a key indicator of gut microbiota imbalance [9]. In general, phenolic-rich extract from various sources holds the potential to module the gut microbiota by adjusting the Firmicutes-to-Bacteroidota (F/B) ratio [33]. In this study, the F/B ratio was highest in the colons of the DSS- treated mice (Figure 5B). After treatment with WPE and SASP, the F/B ratio in the colon of mice within each treatment group was significantly reduced, compared to the DSS group. In a study on DSS-induced UC in mice, He et al. [9] observed a significant decrease in F/B ratio following the administration of STE. This finding is consistent with our observation. Moreover, DSS-induced UC altered the structure of Firmicutes. It markedly upregulated the relative abundance of Firmicutes_A and significantly downregulated the relative abundance of Firmicutes_D. This perturbation in microbial balance was mitigated and reversed by WPE treatment.

At the genus level, principal component analysis (PCA) was employed to reveal intergroup variations in microbiota, with the score plot shown in Figure 5C. The DSS group was clearly distinct from the NC group, whereas the LD, HD, and SASP groups displayed a greater affinity with the NC group in comparison. These results further corroborated the capacity of WPE to modulate the gut microbiota composition in UC mice, thereby approximating it to that of normal mice, aligning with our above findings. Furthermore, *Muribaculum* and *Desulfovibrio* exhibited, to some degree, a negative correlation with DSS- group samples and a positive correlation with NC- group samples, as shown in the loading plot (Figure 5D).

To further explore the differences, the biological classifications of the top 20 genera with the highest relative abundance were presented in Figure 5E, alongside the cladogram and score plots obtained through linear discriminant analysis effect size (LEfSe), depicted in Figure 5F,G. All dominant genera came from the phyla Bacteroidota and Firmicutes, except for *Desulfovibrio*. *Desulfovibrio* had a higher relative abundance in the colon of the NC- group mice (2.01 ± 0.34%), which notably diminished upon DSS administration (0.40 ± 0.08%). Nonetheless, both WPE and SASP treatments were effective in reversing this decline, with 4.97 ± 1.24% in the LD group, 2.73 ± 0.41% in the HD group, and 3.18 ± 0.75% in the SASP group. Though *Desulfovibrio* was commonly regarded as a harmful bacterium in the gut [34], Yi et al. [35] reported that its abundance was negatively correlated with the expression of aspartate aminotransferase and IL-1*β* in mice serum, suggesting a beneficial influence against inflammation. In another study, DSS-induced UC mice showed a decrease in *Desulfovibrio* abundance, which was reversed by fecal microbiota transplantation from normal mice and by Pu-erh tea extract [34], aligning with our findings.

The relative abundance of *Kineothrix* was the highest in DSS-group mice (3.71 ± 0.52%), and significantly higher than that of NC (2.41 ± 0.33%) and SASP (2.56 ± 0.41%) mice, while WPE treatment effectively mitigated this elevation (LD: 1.96 ± 0.28%, HD: 1.57 ± 0.36%). *Kineothrix*, a butyric acid-producing bacterium, has been implicated in the loss of acetic acid and propionic acid metabolism, lipid metabolism disorders, and the heightened risk of intestinal damage due to its increased abundance [36].

*Ligilactobacillus* was another member of the Firmicutes phylum, with a relative abundance of 0.85 ± 0.18% in the NC group. It was significantly downregulated to 0.30 ± 0.10% in the DSS group, while it was upregulated in the LD (1.08 ± 0.28%), HD (3.03 ± 0.69%), and SASP (1.08 ± 0.32%) groups. There was no significant difference among the NC, LD, and SASP groups. *Ligilactobacillus* has potential as a significant probiotic in the gut, with increasing evidence indicating specific species of *Ligilactobacillus*, such as *Ligilactobacillus murinus*, are widely involved in the host’s intestinal metabolism and immune responses, and are closely related to gut health [32]. For example, it has been demonstrated to possess a spectrum of anti-colitis effects, such as upregulating beneficial metabolites, downregulating pro-inflammatory cytokines (TNF-*α* and IL-6), restoring the intestinal mucosal barrier, and enhancing the host’s resistance to DSS-induced UC [32].

Furthermore, the relative abundance of *Duncaniella* in the colons of NC, DSS, and SASP-treated mice (1.75 ± 0.45%, 2.24 ± 0.32%, and 2.90 ± 0.77%, respectively) did not exhibit significant difference, while WPE treatment notably augmented it (LD: 4.25 ± 0.53%, HD: 3.99 ± 0.44%). Current studies on f_Muribaculaceae have mainly focused on its intestinal ecology and taxonomic classification, with a lack of functional potential [37]. Nevertheless, certain genera within this family are thought to possess protective properties against colitis. For example, a particular type of *Duncaniella* has been demonstrated to shield against intestinal epithelial cell damage and afford protection against DSS-induced injury to the host [37]. Similarly, the relative abundance of *Turicibacter* significantly increased following WPE and SASP treatments (LD: 2.04 ± 0.46%, HD: 1.50 ± 0.28%, SASP: 5.64 ± 0.61%), with no significant difference detected between NC and DSS groups, and the relative abundance remained below 0.50%. Liu et al. [14] demonstrated that Fucoidan can enhance the metabolism of SCFAs, particularly butyric acid, by upregulating the abundance of bacteria such as *Turicibacter*, which may ameliorate DSS-induced UC.

Moreover, *Alistipes* is a gut microbial constituent that produces anti-inflammatory metabolites. He et al. [9] found that *Alistipes* positively correlated with the levels of IL-10, butyric acid metabolism, goblet cell count, and TJ protein expression, whereas it negatively correlated with the inflammatory signaling of histone deacetylase 3 (HDAC3)/NF-κB and the expression of IL-1*β* and IL-6. In this study, it was found that the relative abundance of *Alistipes* in the DSS group (0.84 ± 0.26%) was significantly lower than that in NC group (2.87 ± 0.55%). Though the LD (1.24 ± 0.38%) and SASP groups (1.14 ± 0.42%) did not display significant differences to the DSS group, the abundance of *Alistipes* was notably elevated in the HD group (1.65 ± 0.33%).

*Peptococcus*, a differential bacterial constituent, was only abundant in the DSS group (0.14 ± 0.01%), while it was significantly diminished in the NC and all treatment groups, to less than 0.01%. *Peptococcus*, a Gram-positive anaerobic coccus, often coexists with other bacteria and causes inflammation. Upon administering bioactive peptides from ham to DSS-induced UC mice, Xing et al. [38] documented a reduction in the abundance of a variety of pathogenic bacteria, including *Peptococcus*, and a modulation of the gut microbiota structure. *Muribaculum* and *Intestinimonas* were differential bacteria in the NC group. Compared to the NC group (14.97 ± 1.53%), the relative abundance of *Muribaculum* in the DSS group (1.86 ± 0.52%) was significantly lower, and was augmented in the SASP group (2.72 ± 0.64%). However, after WPE treatment, it greatly increased in both the LD and HD groups, reaching 6.69 ± 1.02% and 10.83 ± 1.65%, respectively. *Muribaculum* is recognized as a beneficial gut microbe that is influenced by diet. It is commonly thought to affect host metabolism, including the regulation of immune responses via SCFAs to suppress pneumonia, with evidence suggesting a favorable impact on intestinal homeostasis [14]. *Intestinimonas*, a butyrate acid-producing bacterium, was significantly downregulated in the DSS group (0.10 ± 0.05%), compared to the NC group (2.51 ± 0.30%), while both WPE and SASP interventions effectively augmented its relative abundance (LD: 1.88 ± 0.35%, HD: 1.58 ± 0.42%, SASP: 2.19 ± 0.49%). Gutiérrez-Sarmiento et al. [39] reported that the ‘Ataulfo’ mango diet can significantly upregulate the relative abundance of *Intestinimonas* in a DSS-induced colitis model, which was consistent with our results.

### 3.6. The Effect of WPE on SCFAs Metabolism

The concentrations of seven kinds of SCFAs in the cecal contents were accurately measured via GC-MS, and the results are shown in Table 1. Obviously, acetic acid, propionic acid and butyric acid were the predominant SCFAs, accounting for over 90% of the total SCFAs in all groups. It has been documented that acetic acid, propionic acid, and butyric acid are the primary SCFAs metabolized by mice and human gut microbiota, comprising 90–95% of the total SCFAs, aligning with our results [33]. Compared with the NC group, the three major SCFAs showed a significant decline after DSS administration, with reductions of 22.17% (acetic acid), 34.66% (propionic acid), and 24.02% (butyric acid), resulting in a 22.95% decrease in total SCFAs. This reduction was alleviated by the WPE and SASP treatments. The concentration of acetic acid in each treatment group exhibited a marked increase, with augmentations of 53.45% (LD), 47.66% (HD), and 57.87% (SASP), compared to the DSS group. Similarly, the propionic acid in the LD, HD and SASP groups showed a notable increase relative to the DSS group, with 35.43%, 20.47%, and 67.23%, respectively. Although the butyric acid levels in the LD and SASP groups did not significantly differ from that in the DSS group, it was notably elevated in the HD group, with a 36.41% increase. These changes led to a substantial augmentation of the total SCFA content across each treatment group relative to the DSS group, with enhancements of 36.99% (LD), 36.56% (HD), and 49.30% (SASP). Hong et al. [30] observed a decrease in the concentration of the three major SCFAs in the feces of colitis rats, and successfully restored the levels of acetic acid and butyric acid by the administration of *Aspergillus oryzae*-fermented okara, which was highly consistent with our findings. Moreover, DSS feeding led to a downregulation in valeric acid and an upregulation in isovaleric acid to a certain extent, and WPE and SASP interventions could alleviate or reverse these alterations. SCFAs stem from carbohydrate fermentation through the different metabolic pathways of gut microbes, which can potentially manipulate the ecological balance of gut microbiota [33]. Furthermore, SCFAs are essential substances that can be absorbed and utilized by the body. SCFAs can modulate luminal pH, affect mucin production, preserve intestinal barrier function, play a pivotal role in certain inflammatory responses regulated by MAPK and NF-κB- related signaling pathways via binding to G-protein-coupled receptor 43 (GPR 43) induce the generation of IL-10-producing T cells, and activate the NOD-like receptor family pyrin domain-containing 3 (NLRP3) inflammasome via the GPR109A and GPR43 signaling pathways [30,33]. WPE was demonstrated to modulate the metabolism of SCFAs in DSS-induced UC mice, restoring it to a normal metabolic state, which in turn enhanced the integrity of the intestinal barrier and mitigated the inflammatory response.

### 3.7. Correlation Analysis

**Figure 6:**
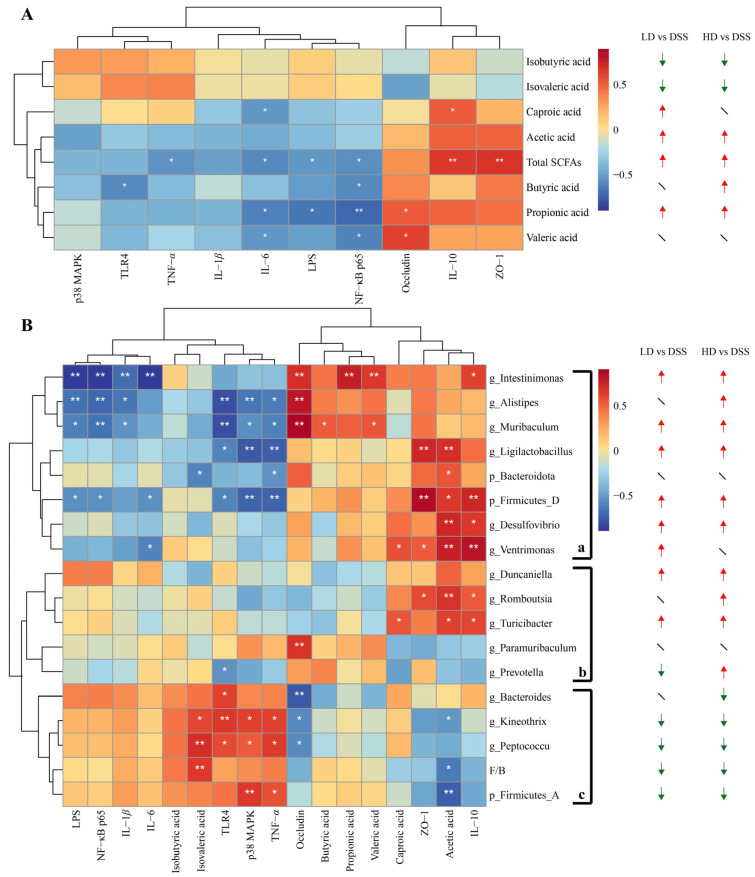
Heatmaps of Spearman’s correlation analysis. (**A**) Correlation between SCFAs and biochemical indicators; (**B**) correlation between gut microbiota with SCFAs and biochemical indicators. * means *p* < 0.05, and ** means *p* < 0.01. The red (or green) arrow indicates a significant increase (or decrease) in the abundance of the corresponding item, compared to the DSS group (*p* < 0.05), whereas the slash indicates no significant difference.

Spearman’s correlation analysis was conducted to further investigate the relationship between biochemical indicators and SCFAs/gut microbiota. As illustrated in the correlation heatmap (Figure 6A), the total SCFAs were positively correlated with IL-10 and ZO-1 (*p* < 0.01), and negatively correlated with TNF-*α*, IL-6, LPS, and NF-κB p65 (*p* < 0.05). These findings suggested that total SCFAs possess a beneficial influence in alleviating colon inflammation and restoring intestinal barrier integrity. Caproic acid showed a positive correlation with IL-10 (*p* < 0.05) and a negative correlation with IL-6 (*p* < 0.05). Butyric acid exhibited a negative correlation with TLR4 and NF-κB p65 (*p* < 0.05). Propionic acid demonstrated a positive correlation with occludin (*p* < 0.05), and negative correlations with NF-κB p65 (*p* < 0.01), IL-6, and LPS (*p* < 0.05). Valeric acid displayed a positive correlation with occludin (*p* < 0.05), and a negative correlation with IL-6 and NF-κB p65 (*p* < 0.05). Despite the lack of significant correlation between acetic acid and biochemical parameters, it was found to have a general positive correlation with IL-10, ZO-1, and occludin and a general negative correlation with pro-inflammatory cytokines and proteins of the TLR4-p38 MAPK/NF-κB signaling pathway. The observed associations indicated that these five SCFAs contributed to the mitigation of UC through various mechanisms. Conversely, isobutyric acid and isovaleric acid did not reveal any significant benefit.

As shown in Figure 6B, the clustering analysis showed the gut microbiota to be categorized into three groups (a–c). Group (a) comprised Bacteroidota and Firmicutes_D at the phylum level, and included *Intestinimonas*, *Alistipes*, *Muribaculum*, *Ligilactobacillus*, and *Desulfovibrio* at the genus level. Clearly, group (a) exhibited a general positive correlation with TJ proteins (occuludin, ZO-1), beneficial SCFAs (butyric acid, propionic acid, valeric acid, capronic acid, acetic acid), and the anti-inflammatory cytokine IL-10. Meanwhile, group (a) was also generally negatively correlated with proteins related to the TLR4-p38 MAPK/NF-κB signaling pathway (NF-κB p65, TLR4, p38 MAPK), pro-inflammatory cytokines (IL-1*β*, IL-6, TNF-*α*), and LPS. These results indicated that the gut microbiota in group (a) have a comprehensive and substantial influence in regulating SCFA metabolism, suppressing the TLR4-p38 MAPK/NF-κB signaling pathway, recovering colon barrier integrity, and mitigating colon inflammation. Moreover, the gut microbiota in group (b) might confer a certain degree of resistance to UC. Specifically, *Romboutsia* exhibited a positive correlation with ZO-1, IL-10 (*p* < 0.05), and acetic acid (*p* < 0.01); *Turicibacter* displayed a positive correlation with capronic acid, acetic acid, and IL-10 (*p* < 0.05); *Paramuribacter* revealed a positive correlation with occludin (*p* < 0.01); *Prevotella* demonstrated a negative correlation with TLR4 (*p* < 0.05). In contrast, the gut microbiota in group (c), which encompassed Firmicures_A, F/B at the phylum level, and *Bacteroids*, *Kineothrix*, and *Peptococcus* at the genus level, predominantly exerted adverse effects on UC. These gut microbes displayed a negative correlation with TJ proteins, beneficial SCFAs, and IL-10, while demonstrating a positive correlation with proteins involved in the TLR4-p38 MAPK/NF-κB signaling pathway, pro-inflammatory cytokines, and LPS. This suggested that these gut microbes may contribute to the exacerbation of inflammation, the disruption of the colon barrier, or the upregulation in the TLR4-p38 MAPK/NF-κB signaling pathway.

**Figure 7:**
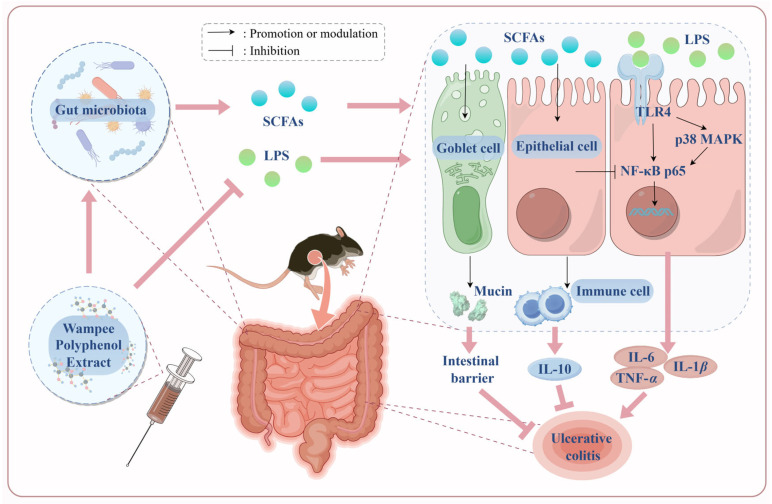
Mechanism of WPE in attenuating DSS-induced UC (figure created using FigDraw).

WPE treatment was observed to selectively and markedly promote the metabolism of the beneficial SCFAs and total SCFAs. Furthermore, WPE treatment generally upregulated the microbial composition in group (a) and group (b), and downregulated the microbial composition in group (c). The above findings have indicated the underlying mechanism by which WPE alleviated UC by inhibiting the LPS-induced TLR4-p38 MAPK/NF-κB signaling pathway and modulating the gut microbiota and its metabolites, as illustrated in Figure 7. Conversely, LPS activates the TLR4 pathway, leading to the phosphorylation of p38 MAPK, which triggers the activation of the transcription factor NF-κB and facilitates the nuclear translocation of the NF-κB p65 subunit, ultimately resulting in the production of inflammatory cytokines such as IL-1*β*, IL-6, and TNF-*α* [27]. On the other hand, SCFAs enhance the secretion of mucin by goblet cells in the intestinal epithelium and reinforce TJs, thereby fortifying the epithelial barrier [40]. Furthermore, specific SCFAs promote the production of the anti-inflammatory cytokine IL-10 in immune cells via GPRs, such as GPR43, and also inhibit the synthesis of pro-inflammatory cytokines by suppressing the HDAC3/NF-κB inflammatory signaling axis [9]. WPE have demonstrated superior therapeutic efficacy in the UC mouse model, primarily through its capacity to impede LPS translocation, regulate gut microbiota composition, and influence SCFA metabolism, as delineated by the aforementioned mechanisms. Although the biotoxicity of WPE remains unknown, it has been reported that the long-term consumption of a diet with quercetin at a concentration of 0.1% could lead to a significant reduction in the life expectancy of mice [41]. While no significant biotoxicity was observed in WPE at either high or low doses in this study, the potential implications for the long-term intake of WPE need to be seriously considered.

## 4. Conclusions

The results in this study demonstrated that WPE promoted weight restoration, diminished DAI, ameliorated various organ indices, and alleviated intestinal epithelial damage. Meanwhile, WPE increased the relative expression of TJ proteins (ZO-1, occludin), downregulated the expression of IL-1*β*, IL-6, TNF-*α*, and LPS in serum, and upregulated the levels of IL-10. Therefore, WPE effectively relieved the symptoms of DSS-induced UC mice by alleviating inflammation, protecting intestinal epithelium, and reinforcing intestinal barrier function. WB analysis revealed that WPE significantly upregulated the expression of key proteins, including TLR4, NF-κB p65, and p38 MAPK. Moreover, 16S rDNA analysis suggested that WPE modulated F/B ratio, enhanced the relative abundances of beneficial bacterial genera (*Muribaculum*, *Ligilactobacillus*, *Intestinimonas*, *Alistipes*, etc.), and diminished the relative abundances of detrimental gut microbes (*Kineothrix*, *Peptococcus*, etc.). These alterations markedly improved intestinal microbial composition and diversity in UC mice. WPE also caused a notable improvement in SCFA metabolism, with the total SCFA level increasing by over 36%. These findings suggest that WPE possessed the capacity to ameliorate SCFA metabolism through regulating the ecological equilibrium of gut microbiota. This regulation of gut microbiota potentially impacted the TLR4-p38 MAPK/NF-κB signaling pathway involved in inflammatory responses. In addition, although high-dose WPE exhibited a more pronounced inhibition of the TLR4-p38 MAPK/NF-κB signaling pathway, it did not demonstrate superior efficacy in UC treatment compared to the lower dose. This may be related to sufficient dosage and treatment duration. This study elucidated the therapeutic efficacy and potential mechanisms of WPE in the DSS-induced UC mice for the first time, thereby providing a theoretical basis for wampee as a potential functional natural food for UC.

## Figures and Tables

**Table 1:** Effect of WPE on metabolic profiles of various SCFAs.

SCFAs (μg/g)	Acetic Acid	Butyric Acid	Isovaleric Acid	Valeric Acid	Caproic Acid	Propionic Acid	Isobutyric Acid	Total
NC	2415.90 ± 237.52 ^b^	408.39 ± 61.81 ^a^	66.18 ± 21.61 ^b^	67.83 ± 13.16 ^ab^	0.77 ± 0.23 ^abc^	513.80 ± 39.36 ^a^	51.25 ± 7.97 ^ab^	3524.12 ± 445.35 ^b^
DSS	1880.23 ± 235.91^c^	310.29 ± 46.52 ^b^	100.86 ± 23.16 ^a^	39.40 ± 6.42 ^c^	0.46 ± 0.15 ^c^	320.30 ± 45.72 ^c^	63.94 ± 9.06 ^a^	2715.49 ± 383.07 ^c^
LD	2885.29 ± 171.01 ^a^	267.11 ± 42.00 ^b^	36.27 ± 7.88 ^c^	53.72 ± 4.69 ^bc^	0.88 ± 0.29 ^ab^	433.78 ± 26.1 ^b^	42.84 ± 4.44 ^bc^	3719.89 ± 205.38 ^ab^
HD	2776.43 ± 167.09 ^a^	423.28 ± 41.84 ^a^	37.53 ± 13.64 ^bc^	47.13 ± 4.79 ^c^	0.66 ± 0.18 ^bc^	385.86 ± 25.46 ^b^	37.39 ± 6.90 ^c^	3708.28 ± 112.68 ^ab^
SASP	2968.38 ± 172.17 ^a^	355.54 ± 81.30 ^ab^	67.06 ± 13.62 ^b^	72.28 ± 15.75 ^a^	1.11 ± 0.32 ^a^	535.65 ± 44.88 ^a^	54.31 ± 9.44 ^ab^	4054.33 ± 276.87 ^a^

The data are expressed as mean ± SD (*n* = 5). The values in the same column with different lowercase letters indicate statistically significant differences (*p* < 0.05).

## Data Availability

The original contributions presented in this study are included in the article/Supplementary Material. Further inquiries can be directed to the corresponding author.

## Supplementary Materials

The following supporting information can be downloaded at: https://www.mdpi.com/article/10.3390/foods14040619/s1, Table S1: TPC, TFC, antioxidant activities, and the contents of major phenolic compounds of WPE.
