# Supplementary material for: Anti-Inflammatory Potential of Wampee (Clausena lansium (Lour.) Skeels) Polyphenol Extract in Ulcerative Colitis: Gut Microbiota and TLR4-p38 MAPK/NF-κB Signaling Axis Regulation
Source: Foods. 2025 Feb 13;14(4):619. doi: 10.3390/foods14040619 (PMC11854079; doi:10.3390/foods14040619)
Supplement: Supplementary file 1 [file foods-14-00619-s001.zip › supplementary material.pdf]

Table S1. TPC, TFC, antioxidant activities, and the contents of major phenolic compounds of WPE

Category	Item (Unit)	Data
General indexes	TPC (mg GAE/g DW)	372.50 ± 13.62
	TFC (mg RE/g DW)	189.39 ± 7.49
Antioxidant activities	DPPH (mM TE/g DW)	13.89 ± 0.53
	FRAP (mM TE/g DW)	14.83 ± 0.48
Major phenolic compounds	Kaempferol (µg/g DW)	37196.89 ± 328.72
	Chlorogenic acid (µg/g DW)	32527.43 ± 348.66
	Isoquercetin (µg/g DW)	32369.78 ± 279.84
	Dihydromyricetin (µg/g DW)	24668.86 ± 210.53
	Quercetin (µg/g DW)	19777.20 ± 263.10
	Luteolin (µg/g DW)	19166.84 ± 166.28
	Rutin (µg/g DW)	18624.70 ± 258.32
	Naringenin (µg/g DW)	13115.64 ± 175.29
	Quercitrin (µg/g DW)	5247.15 ± 68.25
	Myricetin (µg/g DW)	3696.24 ± 51.27

Note: The data are expressed as mean ± SD (n=3).
